# Editorial: Behavior and self-similarity between nano and human scales: from T-pattern and T-string analysis (TPA) with THEME to T-societies

**DOI:** 10.3389/fpsyg.2023.1248233

**Published:** 2023-07-18

**Authors:** Magnus S. Magnusson, Gudberg K. Jonsson, Mariona Portell, M. Teresa Anguera, Maurizio Casarrubea, Frank Schwab

**Affiliations:** ^1^Human Behavior Laboratory, School of Health Sciences, University of Iceland, Reykjavík, Iceland; ^2^Department of Psychobiology and Methodology of Health Sciences, Universitat Autònoma de Barcelona, Cerdanyola del Vallès, Spain; ^3^Faculty of Psychology, Institute of Neurosciences, University of Barcelona, Barcelona, Spain; ^4^Laboratory of Behavioral Physiology, Department of Biomedicine, Neuroscience and Advanced Diagnostics (Bi.N.D.), Human Physiology Section “Giuseppe Pagano”, University of Palermo, Palermo, Italy; ^5^Department of Media Psychology, University of Würzburg, Würzburg, Germany

**Keywords:** self-similarity, TPA, THEME, T-patterns, T-system, T-societies, proteins, mass-societies

This project was inspired in the sixties by primatologist Morris's (1967) “The Naked Ape,” Tinbergen (1963), Von Frisch (1967), and Lorenz (1974) ethological research, rewarded in 1973 by a shared Nobel Prize in Medicine or Physiology, and Wilson's (1975) opus “Sociobiology”. Other important inspirations were Skinner (1969) work on probabilistic real-time contingencies, Chomsky (1957) on syntactic structure and creativity, Montagner (1971) on interactions in social insects and in children (Montagner, 2012), Duncan (1977) on turn-taking in human dyadic interactions, and Dawkins (1976) on behavioral hierarchy and detection algorithms.

Structured animal mass-societies (>10^4^ individuals) are only found in insects and modern humans and understanding their similarities and differences became a major aim through a search for hidden interaction patterns. Existing multivariate and artificial neural network methods and models lacked adequate description and detection of complex real-time patterns requiring new mathematical time structure (1-D) models, now the T-system, with detection algorithms and software (THEME™).

The first was the T-pattern concept, a statistical hierarchical self-similar (pseudo-fractal) pattern recurring with significant translation symmetry since detected in human, animal, and brain network behavior (Magnusson, 1996, 2000, 2005; Casarrubea et al., 2015, 2018; Magnusson et al., 2016; Casarrubea and Di Giovanni, 2020). Gradually, more structural concepts i.e., T-meme, T-composition, T-associate, T-packet, T-music, T-string, T-religion, T-money, and T-society have been added constituting the T-system (Magnusson, 2020).

Spatial string T-patterns, called T-strings, characterize the extra-individual voluminous purely informational (inert) texts essential for the formation of specialized individuals in human mass-societies and the giant extra-individual DNA molecules essential for the formation of citizens in protein mass-societies. Such mass-societies based on Giant Extra-Individual Purely Informational T-strings or GEIPITS, and thus called T-Societies, are only found in proteins and humans and T-Societies of T-Societies only in humans, a unique self-similarity.

T-societies are notably not found in earlier Homo Sapiens nor in social insects that use very different mechanisms, more like those in bodies as societies of cells.

With the invention of writing, powerful precise cumulative extra-individual (external) memory, in a biological eye-blink human T-Societies appeared with this unique self-similarity across some nine orders of magnitude in years and size, coinciding with explosive growth in human knowledge, laws, science, and technology. While humans descend from earlier primates, human mass-societies descend from the far earlier mass-societies of proteins existing on the same bio-mathematical continuum unifying culture and biology.

Presenting a new view of human modern mass social life, we aim to give the reader the most up-to-date perspective on how T-pattern detection and analysis (TPA) with Theme has led to new insight into the structure of behavior, interaction, communication and social organization in both human and non-human subjects. Contributions in this Research Topic (RT) range from different areas such as behavioral and brain sciences, health sciences, sport science, education, and communication thus suggesting the remarkable affordability and versatility of TPA in the study of the most multifaceted aspects of human and non-human behavior.

The diversity of disciplines utilizing TPA does not place a preferential order in the fruition of the contents, with the sole exception of the contribution that the Reader of this RT should take into consideration first, i.e. that of Magnusson, creator of the T-system and TPA. In his elegant work, in fact, the Author presents important propaedeutic aspects of theoretical and conceptual order concerning the model and method at the basis of the detection algorithm. He then continues with the definition of his new and striking addition to the T-system, the concept of T-societies the basis for his definition of the biomathematical self-similarity and uniqueness of human mass-societies at the heart of this RT. The RT continues with a contribution by Fioretti and Neumann in which the Authors summarize and rationalize current typologies of organizational forms, arranging available classifications in a hierarchy of increasing generality; in a second step, they discuss various communication structures pointing to the presence of several sources endowed with global connections as the most efficient diffusion mode. Sáiz-Manzanares et al. present a systematic review of the use of TPA during the last half decade with a special focus on mixed methods and data mining techniques; the contribution offers a detailed overview of the use of the approach in the study of human and non-human behavior. Alonso-Vega et al. discuss the role of verbal interaction pattern analysis in Clinical Psychology, analyze the patterns of verbal interactions, and compare the results using TPA and GSEQ, i.e., a software tool sequential analysis. Simon and Blanchet focus on three adult-child relationships: those with a foster carer, a father, and a mother; interestingly, very different interactive processes were identified: for instance, with the foster carer the interaction patterns were mainly focused on play objects, whereas they involved more collaborative activity with the father and distraction/avoidance behaviors with the mother. Santos et al. discuss the use of TPA in Sport Science, by analyzing offensive and defensive actions of football goalkeepers; the authors argue that the study could contribute to a better knowledge of the goalkeeper's behaviors in the competition. Pennill and Timmers propose an original application of TPA in describing patterns of verbal interaction in newly formed music ensembles; the Authors suggest that such a novel use of TPA could contribute to the understanding of human group behavior and interaction patterns leading to expert team performance. Casarrubea et al. present a review of the use of different approaches, such as transition matrices based analyses and TPA, in the study of the behavior from rodents to non-human primates; the Authors discuss the advantages and drawbacks of each approach and suggest that their work could represent a useful background for all scientists who intend to study quantitative and structural aspects of behavior. Anguera et al. present an elegant review of the use of TPA in the modern scientific literature; following PRISMA guidelines, searches were carried out by means of different and widely known scientific databases such as Scopus, PsycINFO, Web of Science; the Authors conclude that TPA shows a remarkable affordability and applicability and that a noticeable presence TPA in studies using observational methodology is evident. Chacón-Moscoso et al. the so-called “Methodological Quality Scale” (MQS), i.e., a tool with adequate reliability, validity evidence, and metric properties; the Authors suggest that, unlike other existing scales, MQS is easy to apply and methodological quality profiles can be obtained in different areas of intervention and with different types of methods. Finally, in the field of Pedagogical Science, Belza et al. present a study concerning the application of TPA to identify important aspects of the choreography followed by Pikler educators; results show complex patterns and how educators establish interaction with children.

As Editors of this RT, we are delighted to say that it has been a great pleasure to host such different contributions and from such different fields of research, and we hope that the Reader will have the same pleasure in reading these contributions, which, we are sure, will be able to provide useful insights for all those researchers who intend to use TPA in their research.

## Author contributions

All authors listed have made a substantial, direct, and intellectual contribution to the work and approved it for publication.
